# Pediatric Burns – Who Requires Follow-up? A Study of Urban Pediatric Emergency Department Patients

**DOI:** 10.5811/westjem.17984

**Published:** 2024-06-14

**Authors:** Theodore Heyming, Andrea Dunkelman, David Gibbs, Chloe Knudsen-Robbins, John Schomberg, Armin Takallou, Bryan Lara, Brooke Valdez, Victor Joe

**Affiliations:** * Children’s Hospital of Orange County, Orange, California; † University of California Irvine, Department of Emergency Medicine, Irvine, California; ‡ Orange County Global Medical Center, Department of General and Plastic Surgery, Santa Ana, California; § University of Cincinnati College of Medicine, Department of Emergency Medicine, Cincinnati, Ohio; ∥ University of California, Irvine, California; ¶ Oregon Health Science University, School of Medicine, Portland, Oregon; # University of California Irvine, Department of General Surgery, Irvine, California

## Abstract

**Introduction:**

Hundreds of children suffer burn injuries each day, yet care guidelines regarding the need for acute inpatient treatment vs outpatient follow-up vs no required follow-up remain nebulous. This gap in the literature is particularly salient for the emergency clinician, who must be able to rapidly determine appropriate disposition.

**Methods:**

This was a retrospective review of patients presenting to a Level II pediatric trauma center, January 1, 2017–December 31, 2019, and discharged with an International Classification of Diseases, Rev 10, burn diagnosis. We obtained and analyzed demographics, burn characteristics, and follow-up data using univariate and bivariate analysis as well as logistic regression modeling. Patients were stratified into three outcome groups: group 1—patients who underwent emergent evaluation at a burn center or were admitted at their first follow-up appointment; group 2—patients who followed up at a burn center (as an outpatient) or at the emergency department (and were discharged home); and group 3—patients with no known follow-up.

**Results:**

A total of 572 patients were included in this study; 58.9% of patients were 1–5 years of age. Sixty-five patients met group 1 criteria, 189 patients met group 2 criteria, and 318 patients met group 3 criteria. Sixty-five percent of patients met at least one American Burn Association criteria, and 79% of all burns were second-degree burns. Flame and scald burns were associated with increased odds (odds ratio [OR] 1.21, OR 1.12) of group 1 vs group 2 + group 3 (*P* = 0.02, *P* < 0.001). Second/third-degree burns and concern for non-accidental trauma were also associated with increased odds of group 1 vs 2 or 3 (OR = 1.11, 1.35, *P* ≤ 0.001, 0.001, respectively). Scald burns were associated with increased odds of group 2 compared to group 3 (OR 1.11, *P* = 0.04). Second/third degree burns were also associated with increased odds of group 2 vs 3 (OR 1.19, *P* ≤ 0.001).

**Conclusion:**

There were few statistically significant variables strongly associated with group 1 (emergent treatment/admission) vs group 2 (follow-up/outpatient treatment) vs group 3 (no follow- up). However, one notable finding in this study was the association of scald burns with treatment (admission or follow-up) suggesting that the presence of a scald burn in a child may signify to clinicians that a burn center consult is warranted.

Population Health Research Capsule What do we already know about this issue?

*Hundreds of children suffer burn injuries each day, but care guidelines for inpatient admission vs outpatient follow-up or no follow-up remain nebulous.*

What was the research question?

*Are there variables associated with how emergency clinicians refer pediatric burn patients for follow-up?*

What was the major finding of the study?

*Flame and scald burns (OR 1.21), and non-accidental trauma (OR 1.11) had higher odds of evaluation at a burn center (P < 0.001).*

How does this improve population health?

*Referral decisions for pediatric burns is challenging; scald burns often require treatment and should almost always warrant treatment at a burn center.*

## INTRODUCTION

Approximately one US child presents to the emergency department (ED) for a burn injury every six minutes; 10,000 are hospitalized over the course of a year.^1^^,^^2^ Burn injuries, especially in children, carry significant risk of physical and psychological sequelae.^2^^–^^5^ In 2017 alone, ED costs relating to pediatric burns amounted to over $700 million and total hospitalization to over $1.5 billion.^6^ Advances in burn therapy have led to an overall trend toward outpatient management, reducing the risk associated with hospitalization and allowing for more efficient treatment and resource allocation.^7^^,^^8^ However, the process of identifying which patients may be best served by inpatient care vs follow-up outpatient treatment vs discharge home without set follow-up is not well delineated.

The American Burn Association (ABA) has published guidelines regarding transfer/referral to regional burn centers; however, understanding and implementation of these guidelines has varied. Some clinicians have perceived these guidelines as absolute transfer criteria and others as consult/referral criteria.^9^ It is, therefore, unsurprising that transfer/consult/referral practices differ widely, with frequent reports of patients being both under- and over-referred.^10^^,^^11^ Interestingly, Anderson et al found that although most pediatric patients presenting to their institution with burn injuries were low acuity, a majority were admitted, and social factors and transfer status were more strongly associated with admission than burn size or mechanism.^12^ In light of these factors, the documented inconsistency of non-burn center clinician’s evaluation of burns, and the lack of randomized control studies, an expert panel devised updated guidelines in 2020.^13^^–^^16^ Perhaps the most important message from this update is the reframing of the ABA criteria as “consultation guidelines.” There do not otherwise appear to be substantive changes regarding more specific disposition recommendations for pediatric patients.

It is notable that emergency physicians—the clinicians most often tasked with the initial evaluation and decision to contact burn centers—were not included until the third stage of the eDelphi process. The 2020 update also includes recommendations regarding telemedicine. While telemedicine certainly has the potential to transform many aspects of patient care, its use in all patients with potentially deep burns may be prohibitive from a time, technological, legal, and insurance perspective. Clearer standards regarding which patients might benefit most from this process, which ones may be transferred without telemedicine consultation, and which may be discharged home with or without follow-up would likely facilitate ED flow and burn center processes.

Our objective in this study was to describe characteristics of pediatric burn patients directly transferred/admitted to a burn center, patients who followed up, and those who did not follow up. We aimed to identify patient and burn characteristics associated with these three groups to better inform clinician disposition decisions. This three-tiered approach was chosen with the emergency clinician in mind as they must be able to determine which patients require immediate transfer, which may benefit from follow-up, and which patients may be discharged home without need for further evaluation. Our secondary objective was to examine the distribution of patients meeting ABA criteria among these three groups.

## METHODS

This retrospective chart review included patients 0–21 years presenting to the ED of a pediatric Level II trauma center January 1, 2017–December 31, 2019 who were discharged with an International Classification of Diseases, Rev 10 (ICD-10) burn diagnosis (ICD-10 codes may be found in the supplementary materials). We collected data regarding demographics, burn mechanism, burn site, degree of burn, total burn surface area (TBSA), ABA criteria, concern for non-accidental trauma (NAT), and manner of arrival. Concern for NAT was considered to be present if documented in the emergency physician’s or social worker’s note. We collected follow-up data from this institution’s ED as well as the two burn centers serving the surrounding region. Data was abstracted by three trained data abstractors (BL, AT, BV) using a standard operating procedure manual. We collected and managed study data using REDCap electronic data capture tools hosted at Children’s Hospital of Orange County. The REDCap data collection form may be found in the supplementary materials. Charts were identified via a query using ICD-10 diagnosis code (ICD-10 codes T20–31) and ED visit date (January 1, 2017–December 31, 2019) as inclusion parameters. A post-hoc inter-rater reliability (IRR) process was completed wherein a newly trained abstractor used the same standard operating procedure manual to review charts at the main study site. The IRR was analyzed using the Cohen kappa, and all data variables were confirmed as having a Cohen kappa coefficient ranging from 0.870–1.000; 67% of variables reviewed resulted in a Cohen kappa coefficient of 1.000. This study was approved by all study institutions’ institutional review boards. As this was a retrospective chart review subjects were not asked to consent to participate in this study.

We stratified patients into three outcome groups for analysis: group 1 patients representing those likely to require interventions or care best provided by a specialized burn center (as opposed to what may be available at a referring institution or in the outpatient setting); group 2 patients representing those whose wounds likely required further follow-up; and group 3 representing those at lowest risk (ie, those who likely did not need any follow-up). Group 1 included patients who were transferred from the presenting ED directly to one of the two regional burn centers (or their respective EDs) or patients who were admitted at their first follow-up visit. Group 2 included patients who followed up at one of the two regional burn centers (in the ED or clinic) or the presenting ED (for a burn-related visit). Group 3 included patients who were not known to follow up (ie, they did not follow up at either burn center’s clinic or ED or the presenting ED and were not initially transferred to a burn center). Outcomes were defined by disposition, (ie, inclusion in group 1, group 2, or group 3).

### Univariate and Bivariate Statistical Analysis

We measured differences in the distribution of continuous and categorical variables reporting frequency and proportions of categorical variables and mean/standard deviation of continuous variables across outcome groups. The bivariate inferential statistics of the Wilcoxon rank-sum test were used to test the difference in distribution of continuous variables of age and total burn surface area. We used the chi-square test of proportions to test the difference in distribution of categorical variables across groups. These bivariate inferential tests were applied to patients meeting the criteria of in either outcome group 1 or outcome group 2 or lower risk outcome group 3. We conducted missingness analysis on those variables with >10% missing data. The Little test was conducted on all variables meeting this missingness threshold.

### Logistic Regression Models

We used logistic regression models to test the association between demographics/observed clinical variables with the probability of treatment group 1, 2, or 3. Variables that were found to have high correlation or variance inflation using R variance inflation factor measurements (R 4.03) were pruned from the model depending upon a variable’s utility as determined by the study team. As these were full models, we did not apply methods related to backward, forward, or stepwise variable selection.

## RESULTS

### Descriptive Analysis

A total of 572 patients were included in this study; 8.04% of patients were <1 year; 58.9% of patients were 1–5 years; 18% were 5–10 years; 8.74% were 11–15 years; and 6.29% were >15 years. Of all study patients, 48.7% were male, 63.4% were Hispanic, and 73.2% had public insurance (or opted for self-pay). Sixty-five patients were directly transferred to a burn center or admitted at their first follow-up visit (group 1), 189 patients attended at least one follow-up visit (group 2), and 318 patients did not follow up at any of the study institutions (group 3). A total of 372 patients (65%) met at least one ABA criteria. The distribution of characteristics by outcome group is shown in Table 1.

**Table 1.** Distribution of sociodemographic and clinical variables across burn treatment outcome groups.

Patient characteristics	Total n = 572	Group 1, n = 65 (direct transfer to burn center or admitted at first follow-up)	Group 2, n = 189 (patient followed up)	Group 3, n = 318 (patient did not follow up)	*P*-value
Age					0.1
<1	46 (8.04%)	10 (15.3%)	16 (8.46%)	20 (6.28%)	
1–5 years	337 (58.9%)	38 (58.4%)	100 (52.9%)	199 (62.5%)	
5–10 years	103 (18.0%)	9 (13.8%)	44 (23.2%)	50 (15.7%)	
11–15 years	50 (8.74%)	5 (7.69%)	15 (7.93%)	30 (9.43%)	
>15	36 (6.29%)	3 (4.61%)	14 (7.40%)	19 (5.97%)	
Gender					0.01
Male	279 (48.7%)	39 (60.9%)	89 (47.0%)	178 (55.9%)	
Female^*^	292 (51.0%)	25 (38.4%)	100 (52.9%)	140 (44.0%)	
Race					0.38
White	345 (60.3%)	36 (55.3%)	121 (64.0%)	188 (59.1%)	
Non-White	227 (39.6%)	29 (44.6%)	68 (35.9%)	130 (40.8%)	
Ethnicity					0.28
Hispanic	363 (63.4%)	47 (72.3%)	119 (62.9%)	197 (61.9%)	
Non-Hispanic	209 (36.5%)	18 (27.6%)	70 (37.0%)	121 (38.0%)	
Insurance					0.74
Private	153 (26.7%)	10 (15.3%)	152 (80.4%)	257 (80.8%)	
Public/self-pay	419 (73.2%)	55 (84.6%)	37 (19.5%)	61 (19.1%)	
Burn mechanism					<.001
Flame	15 (2.62%)	3 (4.06%)	3 (1.58%)	9 (2.83%)	
Scald	261 (45.6%)	48 (73.8%)	93 (49.2%)	120 (37.7%)	
Steam	6 (1.04%)	0 (0%)	1 (0.52%)	5 (1.57%)	
Chemical	40 (6.99%)	2 (3.07%)	6 (3.17%)	32 (10.0%)	
Electrical	5 (0.87%)	0 (0%)	3 (1.58%)	2 (0.62%)	
Contact	215 (37.5%)	10 (15.3%)	78 (41.2%)	127 (39.9%)	
Other	30 (5.24%)	2 (3.07%)	5 (2.6%)	23 (7.23%)	
Burn site					<.001
Head/neck/face	65 (11.3%)	7 (10.7%)	12 (6.34%)	46 (14.4%)	
Lower limb (Including knees, ankle, foot, sole)	137 (23.9%)	17 (26.1%)	56 (29.6%)	64 (20.1%)	
Perineum/ genitalia	9 (1.57%)	2 (3.07%)	4 (2.11%)	3 (0.94%)	
Trunk/back	87 (15.2%)	15 (23.0%)	20 (10.5%)	52 (16.3%)	
Upper limb (excluding wrist and hand)	82 (14.3%)	13 (20%)	21 (11.1%)	48 (15.0%)	
Wrist/hand/palm	189 (33.0%)	11 (16.9%)	75 (39.6%)	103 (32.3%)	
Missing site	3 (0.52%)	0 (0%)	1 (0.52%)	2 (0.62%)	
Degree of burn					<.001
1^st^	114 (19.9%)	4 (6.15%)	23 (12.1%)	87 (27.3%)	
2^nd^	452 (79.0%)	59 (90.7%)	163 (86.2%)	230 (72.3%)	
3^rd^	6 (1.04%)	2 (3.07%)	3 (1.58%)	1 (0.31%)	
Total burn surface area (TBSA)^**^					<.001
<1%	153 (26.7%)	5 (7.69%)	36 (19.0%)	112 (35.2%)	
1 to 1.9%	20 (3.49%)	0 (0%)	11 (5.82%)	9 (2.83%)	
2 to 4.9%	117 (20.4%)	15 (23.0%)	47 (24.8%)	55 (17.2%)	
5 to 9.9%	42 (7.34%)	21 (32.3%)	11 (5.82%)	10 (3.14%)	
10 to 15%	10 (1.74%)	9 (13.8%)	1 (0.52%)	0 (0%)	
>15%	5 (0.87%)	4 (6.15%)	0 (0%)	1 (0.31%)	
Not stated	225 (39.3%)	11 (16.9%)	83 (43.9%)	131 (41.1%)	
Was ABA referral criteria met?					<.001
Yes	372 (65.0%)	56 (86.1%)	128 (67.7%)	188 (59.1%)	
No	193 (33.7%)	9 (13.8%)	58 (30.6%)	126 (39.6%)	
Not stated	7 (1.22%)	0 (0%)	3 (1.58%)	4 (1.25%)	
Was there concern for non-accidental trauma?					<.001
Yes	52 (9.09%)	15 (23.0%)	17 (8.99%)	20 (6.28%)	
No	520 (90.9%)	50 (76.9%)	172 (91.0%)	298 (93.7%)	

* Gender was recorded as undetermined for one patient.

** Missing TBSA values were significantly associated with outcome group.

*ABA*, American Burn Association.

There was a significant difference associated with gender distribution among groups 1, 2, and 3, with a higher percentage of males in groups 1 and 3 as compared to females, and a higher percentage of females in group 2, *P* = 0.01. There was also a significant difference associated with burn mechanism, with a higher percentage of scald and contact burns than other burn mechanisms in all three groups; scald burns were the predominant burn type in groups 1 and 2 (73.8% and 49.2%, respectively), *P* < 0.001. The location of the burn was also associated with a significant difference between groups 1, 2, and 3, with a predominance of wrist/hand/palmar burns in groups 2 and 3 (39.6% and 32.3%, respectively) compared to lower extremity burns in group 1 (26.1%), *P* ≤ 0.001 (Table 1).

The majority of all burns were second-degree burns (79%). There was a significant difference associated with meeting at least one ABA criteria, with 86.1% of those in group 1 meeting the criteria compared to 67.7% in group 2 and 59.1% in group 3, *P* = 0.01. Concern for NAT was also associated with a significant difference, with 23% of those in group 1 with concern for NAT compared to 8.99% and 6.28% in groups 2 and 3, respectively (*P* ≤ 0.001) (Table 1).

### Logistic Regression Analysis - Group 1 vs 2

Age 5–10 years was associated with decreased odds (odds ratio [OR] 0.86) of direct transfer/admission at first follow-up (group 1), compared to attending at least one follow-up visit (group 2), *P* = 0.04. Flame and scald burns were associated with increased odds (OR 1.52, OR 1.17, respectively) of a group 1 vs 2 outcome, as was concern for NAT (OR 1.48), *P* = 0.02, *P* = 0.02, *P* = 0.02. Head/neck/facial burns, burns to the trunk, and burns to the upper limb (excluding the wrist/hand/palm) were also associated with increased odds of group 1 vs group 2 outcomes (OR 1.26, 1.22, and 1.21, respectively, *P* = 0.04, *P* = 0.04, *P* = 0.04) (Table 2).

**Table 2.** Logistic regression model: estimated odds ratios of group 1 vs group 2.

Patient characteristic	Odds ratio	95% Confidence interval		*P*-value
Age (reference: 1–5 years)				
<1	1.05	0.87	1.26	0.62
5–10 years	0.86	0.75	0.99	0.04
11–15 years	0.93	0.76	1.15	0.52
>15	0.94	0.75	1.18	0.61
Race (reference: non-White)				
White	0.95	0.85	1.07	0.42
Gender (reference: female)				
Male	0.94	0.85	1.05	0.29
Ethnicity (reference: non-Hispanic)				
Hispanic	1.04	0.93	1.17	0.50
Insurance (reference: public insurance)				
Commercial insurance	1.02	0.89	1.18	0.78
Burn mechanism (reference: contact)				
Chemical	1.13	0.81	1.57	0.47
Electrical	0.91	0.56	1.49	0.70
Flame	1.52	1.06	2.19	0.02
Other	1.09	0.78	1.52	0.61
Scald	1.17	1.03	1.33	0.02
Steam	0.74	0.32	1.71	0.48
Degree of burn (reference: 1st degree)				
2nd degree or 3rd degree	1.12	0.93	1.35	0.22
Burn site (reference: wrist/hand/palm)				
Head/neck/face	1.26	1.02	1.56	0.04
Lower limb (knees, ankle, foot, sole)	1.05	0.91	1.21	0.51
Perineum/genitalia	1.17	0.81	1.67	0.40
Trunk	1.22	1.01	1.47	0.04
Upper limb	1.21	1.01	1.45	0.04
Was there concern for non-accidental trauma? (Reference: No)				
Yes	1.48	1.07	2.06	0.03

### Group 1 vs Group 3

Male gender was associated with decreased odds of direct transfer/admission at first follow-up (group 1) compared to not following up (group 3) (OR 0.92, *P* = 0.02). Scald burns were associated with increased odds (OR 1.23, of group 1 vs group 3 outcomes, *P* < 0.001). Second/third degree burns and concern for NAT were also associated with increased odds of group 1 vs group 3 outcomes (OR 1.21 and 1.49, respectively, *P* < 0.001, *P* = 0.003) (Table 3).

**Table 3.** Logistic regression model: estimated odds ratios group 1 vs group 3.

Patient characteristics	Odds ratio	95% Confidence interval		*P*-value
Age (reference: 1–5 years)				
<1	1.10	0.96	1.27	0.18
5–10 years	0.96	0.86	1.07	0.46
11–15 years	1.04	0.91	1.19	0.58
>15	1.03	0.88	1.20	0.75
Race (reference: non-White)				
White	1.00	0.93	1.08	0.90
Gender (reference: female)				
Male	0.92	0.85	0.98	0.02
Ethnicity (reference: non-Hispanic)				
Hispanic	1.06	0.98	1.15	0.13
Insurance (reference: public insurance)				
Commercial insurance	0.99	0.91	1.09	0.91
Burn mechanism (reference: contact)				
Chemical	1.05	0.88	1.26	0.57
Electrical	1.04	0.64	1.71	0.86
Flame	1.23	0.99	1.53	0.07
Other	0.94	0.81	1.10	0.44
Scald	1.23	1.13	1.35	<.001
Steam	0.88	0.63	1.21	0.43
Degree of burn (reference: 1st degree)				
2nd degree or 3rd degree	1.21	1.10	1.34	<.001
Burn site (reference: wrist/hand/palm)				
Head/neck/face	1.11	0.96	1.28	0.16
Lower limb (knees, ankle, foot, sole)	1.02	0.92	1.13	0.75
Perineum/genitalia	1.23	0.89	1.71	0.22
Trunk	1.01	0.89	1.13	0.91
Upper limb	1.07	0.96	1.21	0.22
Was there concern for non-accidental trauma? (Reference: No)				
Yes	1.49	1.15	1.93	<0.001

### Group 2 vs Group 3

Scald burns were associated with increased odds of follow-up (group 2) compared to no follow-up (group 3) (OR 1.11, *P* = 0.04). Second/third degree burns were also associated with increased odds of group 2 vs group 3 outcomes (OR 1.19, *P* ≤ .0001). Burns to the trunk were associated with decreased odds of group 2 vs group 3 outcomes (OR 0.81, *P* ≤ .0001) (Table 4).

**Table 4.** Logistic regression model results: estimated odds ratios of group 2 vs group 3.

Patient characteristics	Odds ratio	95% Confidence interval		*P*-value
Age (reference: 1–5 years)				
<1	1.06	0.90	1.26	0.48
5–10 years	1.10	0.98	1.24	0.09
11–15 years	1.02	0.87	1.20	0.82
>15	1.09	0.91	1.30	0.36
Race (reference: non-White)				
White	1.07	0.98	1.17	0.14
Gender (reference: female)				
Male	0.93	0.85	1.01	0.09
Ethnicity (reference: non-Hispanic)				
Hispanic	1.00	0.92	1.10	0.95
Insurance (reference: public insurance)				
Commercial insurance	1.03	0.92	1.14	0.66
Burn mechanism (reference: contact)				
Chemical	0.98	0.78	1.22	0.83
Electrical	1.19	0.78	1.81	0.43
Flame	0.95	0.71	1.27	0.72
Other	0.86	0.71	1.04	0.12
Scald	1.11	1.00	1.23	0.04
Steam	0.89	0.59	1.32	0.55
Degree of burn (reference: 1st degree)				
2nd degree or 3rd degree	1.19	1.06	1.33	<0.001
Burn site (reference: wrist/hand/palm)				
Head/neck/face	0.90	0.75	1.07	0.23
Lower limb (knees, ankle, foot, sole)	0.98	0.87	1.11	0.76
Perineum/genitalia	1.13	0.78	1.62	0.52
Trunk	0.81	0.70	0.94	<0.001
Upper limb	0.88	0.77	1.01	0.08
Was there concern for non-accidental trauma? (Reference: No)				
Yes	1.10	0.79	1.54	0.57

### Group 1 or 2 vs Group 3

Male gender was associated with decreased odds of direct transfer/admission at first follow-up (group 1), or any follow-up (group 2) compared to not following up (group 3) (OR 0.904, *P* = 0.01.) Scald burns and second/third degree burns were associated with group 1 or 2 outcomes vs group 3 outcomes (OR 1.18 and 1.261, respectively, *P* ≤ 0.001, *P* < 0.001). Burns to the trunk were associated with decreased odds (OR 0.857, of group 1 or 2 outcomes compared to group 3, *P* = 0.03) (Table 5).

**Table 5.** Logistic regression model: estimated odds ratios of group 1 or 2 vs group 3.

Patient characteristics	Odds ratio	95% Confidence interval		*P*-value
Age (Reference: 1–5 years)				
<1	1.091	0.935	1.272	0.27
5–10 years	1.062	0.951	1.186	0.29
11–15 years	1.025	0.881	1.193	0.75
>15	1.094	0.923	1.297	0.30
Race (reference: non-White)				
White	1.056	0.972	1.148	0.20
Gender (reference: female)				
Male	0.904	0.833	0.980	0.01
Ethnicity (reference: non-Hispanic)				
Hispanic	1.024	0.939	1.117	0.59
Insurance (reference: public insurance)				
Commercial insurance	1.022	0.920	1.136	0.68
Burn mechanism (reference: contact)				
Chemical	0.985	0.801	1.211	0.88
Electrical	1.169	0.763	1.790	0.47
Flame	1.064	0.818	1.383	0.64
Other	0.839	0.695	1.014	0.07
Scald	1.180	1.070	1.302	<0.001
Steam	0.820	0.549	1.223	0.33
Degree of burn (reference: 1st degree)				
2nd degree or 3rd degree	1.261	1.127	1.411	<0.001
Burn site (reference: wrist/hand/palm)				
Head/neck/face	0.949	0.805	1.118	0.53
Lower limb (knees, ankle, foot, sole)	0.986	0.881	1.104	0.81
Perineum/genitalia	1.183	0.853	1.640	0.32
Trunk	0.857	0.748	0.982	0.03
Upper limb	0.932	0.818	1.062	0.29
Was there concern for non-accidental trauma? (Reference: No)				
Yes	1.277	0.961	1.698	0.09

### Group 1 vs 2 or 3

Flame and scald burns were associated with increased odds of direct transfer/admission at first follow-up (group 1) vs following up at least once (group 2) or not following up (group 3) (OR 1.21 and 1.12, *P* = 0.02, *P* < 0.001). Second/third degree burns and concern for NAT were also associated with increased odds of group 1 vs 2 or 3 outcomes (OR 1.11, 1.35, respectively, *P* ≤ 0.001, 0.001) (Table 6).

**Table 6.** Logistic regression model - estimated odds ratios of group 1 vs Group 2 or 3.

Patient characteristic	Odds ratio	95% Confidence interval		*P*-value
Age (reference: 1–5 years)				
<1	1.06	0.96	1.17	0.22
5–10 years	0.95	0.88	1.02	0.15
11–15 years	1.00	0.91	1.10	0.97
>15	1.01	0.90	1.12	0.90
Race (reference: non-White)				
White	0.98	0.93	1.04	0.56
Gender (reference: female)				
Male	0.95	0.90	1.00	0.05
Ethnicity (reference: non-Hispanic)				
Hispanic	1.04	0.98	1.10	0.19
Insurance (reference: public insurance)				
Commercial insurance	1.00	0.94	1.07	0.94
Burn mechanism (reference: contact)				
Chemical	1.02	0.90	1.17	0.73
Electrical	0.97	0.74	1.27	0.82
Flame	1.21	1.03	1.44	0.02
Other	0.96	0.85	1.08	0.51
Scald	1.12	1.06	1.20	<0.001
Steam	0.90	0.70	1.16	0.41
Degree of burn (reference: 1st degree)				
2nd degree or 3rd degree	1.11	1.04	1.20	<0.001
Burn site (reference: wrist/hand/palm)				
Head/neck/face	1.09	0.98	1.21	0.10
Lower limb (knees, ankle, foot, sole)	1.01	0.94	1.09	0.79
Perineum/genitalia	1.12	0.91	1.38	0.28
Trunk	1.05	0.96	1.15	0.27
Upper limb	1.08	0.99	1.17	0.07
Was there concern for non-accidental trauma? (Reference: No)				
Yes	1.35	1.12	1.62	<0.001

## DISCUSSION

In this retrospective study we attempted to describe the population of pediatric patients presenting to our ED with burn injuries as well as investigate whether there may be patient or burn characteristics associated with particular outcomes. Our study population reflected national statistics with regard to burn mechanism with a predominance of scald (45.6%) and contact burns (37.5%).^17^ This appears similar to an Australian study by Abeyasundara et al in which the majority of burns were scald, followed by contact.^18^ It is, however, slightly different from work by Abramowicz et al who examined pediatric visits to the ED (using the Nationwide Emergency Department Sample database) for burn-related injuries and reported that a majority of burns were due to electrical appliances, followed by scald injuries.^6^ Scald burns were generally associated with need for treatment, both in our study (increased ORs of group 1 or group 2 outcomes) and in analysis by Mitchell et al, which demonstrated an almost three-fold increase in likelihood of admission for patients with scald burns compared to other burn mechanisms.^1^ These population findings are especially important when considering local injury prevention and education efforts.

The majority of patients in our study (58.9%) were between the ages of 1–5. This data is similar to that reported by Abeyasundara et al who found that children between the ages of 1–5 years of age accounted for 59.3% of all children (0–16 years of age) in their study.^18^ This is likely reflective of developmental abilities achieved (and lacking) during this period. In addition, the large percentage of patients 1–5 years in group 3 (62.5%) is perhaps indicative of the increased mobility of these children coupled with increased parental concern for burns in younger children.

Interestingly, and in contrast to other studies, 51% of patients in our study were female, whereas Mitchell et al who analyzed the US National Electronic Injury Surveillance system from 1990–2014 and Abramowicz et al who examined the Nationwide Emergency Department Sample from 2008–2013, found a majority of patients were male (58.4% and 56%, respectively).^1^^,^^6^ Of note, however, the majority of patients in group 1 (likely representing the most serious burns) and group 3 (those who didn’t follow up) were male compared to group 2 in which the majority were female. One possible explanation for this discrepancy is increased parental concern in our population for burn injuries in females as compared to males.

In this study we examined rates of transfer to a burn center and admission at the first follow-up visit (group 1). Eleven percent of patients in this study fell into this category, similar to admission rates reported by Mitchell et al and Abramowicz et al.^1^^,^^6^ In addition, we analyzed transfer/admission rates and follow-up by ABA criteria. Among those in group 1, 86.1% met ABA criteria; however, 67.7% of those in group 2 met criteria, and 59.1% of those in group 3 even met ABA criteria. Although the ABA guidelines are meant to assist in building an appropriate referral system and not meant to be definitive care recommendations, our data suggests that adaptations to the ABA criteria may be valuable as many children, including those who don’t seem to require follow-up care, meet current ABA guidelines. Further research regarding this low-risk population would likely benefit both EDs and burn referral centers.

Several studies have shown there is confusion and differing policies regarding ABA guidelines and the need for referral vs transfer vs specialist consult.^10^ For example, Johnson et al reported that only 8.2% of pediatric burn patients meeting ABA transfer guidelines were transferred from low-volume hospitals, Doud et al reported an under-referral rate of 55%, and Van Yperen et al found that according to the referral criteria of the Australian Emergency Management of Severe Burns course, just over 25% of patients (adult and pediatric) were under-transferred.^19^^–^^21^ However, Rose et al examined the referral patterns of children presenting to an ED in the United Kingdom (UK) for burn injuries and reported that although 74% were under-referred only 3.2% of these patients subsequently required referral to a burn unit and none required specialist intervention, suggesting that complete adherence to the UK’s burn referral criteria (National Burn Care Review) might not be necessary and in fact might necessarily increase the workload of regional burn units.^22^ Notably, Garcia et al examined admission practices at 34 pediatric burn centers across the US and found significant variation in admission decisions regarding patients with minor burns (<10% TBSA) vs ED-initiated outpatient management.^11^ In this setting of significant practice variation in multiple countries, and lack of definitive guidance regarding best practices, we attempted to identify which characteristics were most associated with admission/transfer or follow-up alone.

Burns to the head/neck/face, trunk, and upper limb were all associated with statistically significantly increased odds of direct transfer/admission at first follow-up compared to attending at least one follow-up visit. Few variables were associated with statistically significant odds of group 2 vs group 3 outcomes. Notably, scald was associated with increased odds of group 2 vs group 3 outcomes. It is not surprising that the presence of second/third degree burns was almost always associated with significantly increased odds of admission or follow-up compared to no follow-up. Concern for NAT was found to be associated with increased odds of group 1 vs 2 or 3 outcomes; however, given the additional considerations necessary when there is concern for NAT, it is difficult to disentangle the social vs clinical considerations behind the ramifications of this finding.

## LIMITATIONS

Limitations of this study include its relatively small sample size and, therefore, limited power and limited generalizability. It is important to note that in this study we used the outcome of admission or follow-up as a proxy for requirement of admission and/or follow-up. In addition, investigator knowledge of follow-up was limited to patients returning either to the ED of initial presentation or to the two regional burn centers. It is possible that some patients in group 3 followed up at outside institutions or primary care clinics. However, the pediatric ED involved in the study is the only pediatric-specific ED in the study county, and the two regional burn centers are the only burn specialty centers in the study county. We did not include length of stay for patients who were directly transferred in this analysis, and it is possible that patients who were directly transferred but discharged from the burn center ED were incorrectly apportioned to group 1. This may have led to characteristics incorrectly associated with need for direct transfer.

## CONCLUSION

This study demonstrates the importance of individual institution/regional population data as it may differ from national estimations, and these statistics may inform injury prevention education and outreach regarding pediatric burns. The limited statistically significant data associated with transfer/admission vs follow-up vs no follow-up was surprising yet illuminates potential causes for the diverse transfer/admission practices demonstrated in previous studies. These results highlight the potential role of telemedicine for expert guidance; however, future studies are necessary to determine which patients may be best suited to telemedicine consults. One notable finding in this study was the association of scald burns with treatment (admission or follow-up), suggesting that the presence of a scald burn in a child may signify to clinicians that a burn center consult is warranted. Future research could expand on this work by analyzing larger patient populations and expanding burn and patient variables to capture further significant data points that may help improve clinician disposition decisions.

## Supplementary Information




