# Supplementary material for: Pediatric Burns – Who Requires Follow-up? A Study of Urban Pediatric Emergency Department Patients
Source: West J Emerg Med. 2024 Jun 14;25(4):634–44. doi: 10.5811/westjem.17984 (PMC11254158; doi:10.5811/westjem.17984)
Supplement: Supplementary file 1 [file wjem-25-634-s001.docx]

**Supplementary Material:**  International Classification of Diseases (ICD) -10 Codes

| **ICD code** | **Description** |
| --- | --- |
| T20.00XA | Burn of unspecified degree of head, face, and neck, unspecified site, initial encounter |
| T20.04XA | Burn of unspecified degree of nose (septum), initial encounter |
| T20.07XA | Burn of unspecified degree of neck, initial encounter |
| T20.12XA | Burn of first degree of lip(s), initial encounter |
| T20.15XA | Burn of first degree of scalp [any part], initial encounter |
| T20.16XA | Burn of first degree of forehead and cheek, initial encounter |
| T20.19XA | Burn of first degree of multiple sites of head, face, and neck, initial encounter |
| T20.25XA | Burn of second degree of scalp [any part], initial encounter |
| T20.29XA | Burn of second degree of multiple sites of head, face, and neck, initial encounter |
| T20.44XA | Corrosion of unspecified degree of nose (septum), initial encounter |
| T20.45XA | Corrosion of unspecified degree of scalp [any part], initial encounter |
| T20.52XA | Corrosion of first degree of lip(s), initial encounter |
| T21.01XA | Burn of unspecified degree of chest wall, initial encounter |
| T21.02XA | Burn of unspecified degree of abdominal wall, initial encounter |
| T21.04XA | Burn of unspecified degree of lower back, initial encounter |
| T21.05XA | Burn of unspecified degree of buttock, initial encounter |
| T21.06XA | Burn of unspecified degree of male genital region, initial encounter |
| T21.07XA | Burn of unspecified degree of female genital region, initial encounter |
| T21.09XA | Burn of unspecified degree of other site of trunk, initial encounter |
| T21.11XA | Burn of first degree of chest wall, initial encounter |
| T21.12XA | Burn of first degree of abdominal wall, initial encounter |
| T21.13XA | Burn of first degree of upper back, initial encounter |
| T21.14XA | Burn of first degree of lower back, initial encounter |
| T21.16XA | Burn of first degree of male genital region, initial encounter |
| T21.20XA | Burn of second degree of trunk, unspecified site, initial encounter |
| T21.21XA | Burn of second degree of chest wall, initial encounter |
| T21.22XA | Burn of second degree of abdominal wall, initial encounter |
| T21.23XD | Burn of second degree of upper back, subsequent encounter |
| T21.24XA | Burn of second degree of lower back, initial encounter |
| T21.25XA | Burn of second degree of buttock, initial encounter |
| T21.26XA | Burn of second degree of male genital region, initial encounter |
| T21.27XA | Burn of second degree of female genital region, initial encounter |
| T21.42XA | Corrosion of unspecified degree of abdominal wall, initial encounter |
| T21.42XD | Corrosion of unspecified degree of abdominal wall, subsequent encounter |
| T21.47XA | Corrosion of unspecified degree of female genital region, initial encounter |
| T22.00XA | Burn of unspecified degree of shoulder and upper limb, except wrist and hand, unspecified site, initial encounter |
| T22.011A | Burn of unspecified degree of right forearm, initial encounter |
| T22.012A | Burn of unspecified degree of left forearm, initial encounter |
| T22.022A | Burn of unspecified degree of left elbow, initial encounter |
| T22.051A | Burn of unspecified degree of right shoulder, initial encounter |
| T22.052A | Burn of unspecified degree of left shoulder, initial encounter |
| T22.059A | Burn of unspecified degree of unspecified shoulder, initial encounter |
| T22.062A | Burn of unspecified degree of left scapular region, initial encounter |
| T22.092A | Burn of unspecified degree of multiple sites of left shoulder and upper limb, except wrist and hand, initial encounter |
| T22.10XA | Burn of first degree of shoulder and upper limb, except wrist and hand, unspecified site, initial encounter |
| T22.111A | Burn of first degree of right forearm, initial encounter |
| T22.131A | Burn of first degree of right upper arm, initial encounter |
| T22.151A | Burn of first degree of right shoulder, initial encounter |
| T22.20XA | Burn of second degree of shoulder and upper limb, except wrist and hand, unspecified site, initial encounter |
| T22.211A | Burn of second degree of right forearm, initial encounter |
| T22.211D | Burn of second degree of right forearm, subsequent encounter |
| T22.212A | Burn of second degree of left forearm, initial encounter |
| T22.221A | Burn of second degree of right elbow, initial encounter |
| T22.222A | Burn of second degree of left elbow, initial encounter |
| T22.241A | Burn of second degree of right axilla, initial encounter |
| T22.259A | Burn of second degree of unspecified shoulder, initial encounter |
| T22.292A | Burn of second degree of multiple sites of left shoulder and upper limb, except wrist and hand, initial encounter |
| T22.30XA | Burn of third degree of shoulder and upper limb, except wrist and hand, unspecified site, initial encounter |
| T22.342A | Burn of third degree of left axilla, initial encounter |
| T22.412A | Corrosion of unspecified degree of left forearm, initial encounter |
| T23.001A | Burn of unspecified degree of right hand, unspecified site, initial encounter |
| T23.002A | Burn of unspecified degree of left hand, unspecified site, initial encounter |
| T23.002D | Burn of unspecified degree of left hand, unspecified site, subsequent encounter |
| T23.009A | Burn of unspecified degree of unspecified hand, unspecified site, initial encounter |
| T23.021A | Burn of unspecified degree of single right finger (nail) except thumb, initial encounter |
| T23.029A | Burn of unspecified degree of unspecified single finger (nail) except thumb, initial encounter |
| T23.032A | Burn of unspecified degree of multiple left fingers (nail), not including thumb, initial encounter |
| T23.062A | Burn of unspecified degree of back of left hand, initial encounter |
| T23.072A | Burn of unspecified degree of left wrist, initial encounter |
| T23.091D | Burn of unspecified degree of multiple sites of right wrist and hand, subsequent encounter |
| T23.101A | Burn of first degree of right hand, unspecified site, initial encounter |
| T23.102A | Burn of first degree of left hand, unspecified site, initial encounter |
| T23.122A | Burn of first degree of single left finger (nail) except thumb, initial encounter |
| T23.131A | Burn of first degree of multiple right fingers (nail), not including thumb, initial encounter |
| T23.172A | Burn of first degree of left wrist, initial encounter |
| T23.201A | Burn of second degree of right hand, unspecified site, initial encounter |
| T23.202A | Burn of second degree of left hand, unspecified site, initial encounter |
| T23.212A | Burn of second degree of left thumb (nail), initial encounter |
| T23.219A | Burn of second degree of unspecified thumb (nail), initial encounter |
| T23.221A | Burn of second degree of single right finger (nail) except thumb, initial encounter |
| T23.222A | Burn of second degree of single left finger (nail) except thumb, initial encounter |
| T23.229A | Burn of second degree of unspecified single finger (nail) except thumb, initial encounter |
| T23.231A | Burn of second degree of multiple right fingers (nail), not including thumb, initial encounter |
| T23.231D | Burn of second degree of multiple right fingers (nail), not including thumb, subsequent encounter |
| T23.232A | Burn of second degree of multiple left fingers (nail), not including thumb, initial encounter |
| T23.241A | Burn of second degree of multiple right fingers (nail), including thumb, initial encounter |
| T23.242A | Burn of second degree of multiple left fingers (nail), including thumb, initial encounter |
| T23.251A | Burn of second degree of right palm, initial encounter |
| T23.252A | Burn of second degree of left palm, initial encounter |
| T23.259A | Burn of second degree of unspecified palm, initial encounter |
| T23.261A | Burn of second degree of back of right hand, initial encounter |
| T23.262A | Burn of second degree of back of left hand, initial encounter |
| T23.271A | Burn of second degree of right wrist, initial encounter |
| T23.291A | Burn of second degree of multiple sites of right wrist and hand, initial encounter |
| T23.292A | Burn of second degree of multiple sites of left wrist and hand, initial encounter |
| T23.332A | Burn of third degree of multiple left fingers (nail), not including thumb, initial encounter |
| T23.469A | Corrosion of unspecified degree of back of unspecified hand, initial encounter |
| T23.509A | Corrosion of first degree of unspecified hand, unspecified site, initial encounter |
| T23.691A | Corrosion of second degree of multiple sites of right wrist and hand, initial encounter |
| T24.001A | Burn of unspecified degree of unspecified site of right lower limb, except ankle and foot, initial encounter |
| T24.002A | Burn of unspecified degree of unspecified site of left lower limb, except ankle and foot, initial encounter |
| T24.009A | Burn of unspecified degree of unspecified site of unspecified lower limb, except ankle and foot, initial encounter |
| T24.011A | Burn of unspecified degree of right thigh, initial encounter |
| T24.012A | Burn of unspecified degree of left thigh, initial encounter |
| T24.019A | Burn of unspecified degree of unspecified thigh, initial encounter |
| T24.032A | Burn of unspecified degree of left lower leg, initial encounter |
| T24.039A | Burn of unspecified degree of unspecified lower leg, initial encounter |
| T24.091A | Burn of unspecified degree of multiple sites of right lower limb, except ankle and foot, initial encounter |
| T24.101A | Burn of first degree of unspecified site of right lower limb, except ankle and foot, initial encounter |
| T24.102A | Burn of first degree of unspecified site of left lower limb, except ankle and foot, initial encounter |
| T24.111A | Burn of first degree of right thigh, initial encounter |
| T24.132A | Burn of first degree of left lower leg, initial encounter |
| T24.201A | Burn of second degree of unspecified site of right lower limb, except ankle and foot, initial encounter |
| T24.202A | Burn of second degree of unspecified site of left lower limb, except ankle and foot, initial encounter |
| T24.209A | Burn of second degree of unspecified site of unspecified lower limb, except ankle and foot, initial encounter |
| T24.211D | Burn of second degree of right thigh, subsequent encounter |
| T24.212A | Burn of second degree of left thigh, initial encounter |
| T24.219A | Burn of second degree of unspecified thigh, initial encounter |
| T24.221A | Burn of second degree of right knee, initial encounter |
| T24.232A | Burn of second degree of left lower leg, initial encounter |
| T24.292D | Burn of second degree of multiple sites of left lower limb, except ankle and foot, subsequent encounter |
| T24.411A | Corrosion of unspecified degree of right thigh, initial encounter |
| T25.012A | Burn of unspecified degree of left ankle, initial encounter |
| T25.021A | Burn of unspecified degree of right foot, initial encounter |
| T25.022A | Burn of unspecified degree of left foot, initial encounter |
| T25.031A | Burn of unspecified degree of right toe(s) (nail), initial encounter |
| T25.121A | Burn of first degree of right foot, initial encounter |
| T25.122A | Burn of first degree of left foot, initial encounter |
| T25.132A | Burn of first degree of left toe(s) (nail), initial encounter |
| T25.192A | Burn of first degree of multiple sites of left ankle and foot, initial encounter |
| T25.211A | Burn of second degree of right ankle, initial encounter |
| T25.212A | Burn of second degree of left ankle, initial encounter |
| T25.221A | Burn of second degree of right foot, initial encounter |
| T25.221D | Burn of second degree of right foot, subsequent encounter |
| T25.221S | Burn of second degree of right foot, sequela |
| T25.222A | Burn of second degree of left foot, initial encounter |
| T25.222D | Burn of second degree of left foot, subsequent encounter |
| T26.00XA | Burn of unspecified eyelid and periocular area, initial encounter |
| T26.02XA | Burn of left eyelid and periocular area, initial encounter |
| T26.10XA | Burn of cornea and conjunctival sac, unspecified eye, initial encounter |
| T26.40XA | Burn of unspecified eye and adnexa, part unspecified, initial encounter |
| T26.41XA | Burn of right eye and adnexa, part unspecified, initial encounter |
| T26.50XA | Corrosion of unspecified eyelid and periocular area, initial encounter |
| T26.51XA | Corrosion of right eyelid and periocular area, initial encounter |
| T26.60XA | Corrosion of cornea and conjunctival sac, unspecified eye, initial encounter |
| T26.61XA | Corrosion of cornea and conjunctival sac, right eye, initial encounter |
| T26.62XA | Corrosion of cornea and conjunctival sac, left eye, initial encounter |
| T26.82XA | Corrosions of other specified parts of left eye and adnexa, initial encounter |
| T26.90XA | Corrosion of unspecified eye and adnexa, part unspecified, initial encounter |
| T28.0XXA | Burn of mouth and pharynx, initial encounter |
| T28.1XXA | Burn of esophagus, initial encounter |
| T28.3XXA | Burn of internal genitourinary organs, initial encounter |
| T28.5XXA | Corrosion of mouth and pharynx, initial encounter |
| T28.6XXA | Corrosion of esophagus, initial encounter |
| T32.0 | Corrosions involving less than 10% of body surface |
